# Prevalence of Obesity in Inguinal Hernia Repair Patients in a Tertiary Care Center

**DOI:** 10.31729/jnma.5636

**Published:** 2021-02-28

**Authors:** Sundar Shrestha, Pramod Kumar Upadhyay

**Affiliations:** 1 Department of General Surgery, Bir hospital, NAMS, Kathmandu, Nepal

**Keywords:** *body mass index*, *inguinal hernia repair*, *obesity*

## Abstract

**Introduction:**

Inguinal hernia is a common surgical problem, with a lifetime risk of 27% in men and 3% in women. Its cumulative incidence is 17.2% and 12.3% in body mass index of <25 kg/m^2^ and 25-30 kg/m^2^ respectively. Obesity had been regarded as the risk factor for the development of an inguinal hernia. However, recent epidemiologic studies have suggested the decreased prevalence of inguinal hernia in increased weight and body mass index individuals. The aim of this study is to find out the prevalence of obesity in inguinal hernia repair patients in a tertiary care center.

**Methods:**

A descriptive cross-sectional observational study was performed in Bir Hospital from May 2018 to December 2019 after taking ethical approval from the institutional review board of NAMS. Convenient sampling was done with a sample size of 219. Statistical analysis was done using SPSS ver. 23 and Microsoft Excel software by descriptive statistics.

**Results:**

The mean body mass index was 22.10 ±3.07 kg/m^2^. Body mass index Category 18.5 - 22.9 kg/m^2^ had 133 (61%) male and seven (3.2%) female patients, category ≥30 kg/m^2^ had four (1.8%) male. Most of inguinal hernia repair patients were farmers 158 (72.5%).Common risk factors noted were smoking 142 (65.1%), heavy work 112 (51.4%), chronic cough 65 (29.8%). Most of the complications occurred in the normal body mass index category and the prevalence of complications decreased as the body mass index increased. The recurrence was found in three (1.4%) inguinal hernia repairs.

**Conclusions:**

The majority of inguinal hernia repair patients were non-obese, and complications were less in obese patients.

## INTRODUCTION

Inguinal hernia (IH) is a common surgical problem. It represents 75% of abdominal wall hernias, with a lifetime risk of 27% in men and 3% in women.^1^ The 20-year cumulative incidence was 17.2% in Body mass index (BMI) of <25 kg/m^2^ and 12.3% in both BMI of 25-30 kg/m^2^, and >30 kg/m^2^.^2^

Increasing age, male gender, smoking, family history, connective tissue disorders, and increased intra-abdominal pressure (IAP) are predisposing factors to develop IH.^3^ Due to increased IAP associated with obesity, it is considered a risk factor for IH.^4,5^ However, recent epidemiologic studies have suggested the opposite.^2,4,5^ By knowing the distribution of BMI in Inguinal hernia repair (IHR) patients, we can identify its outcome in postoperative complications, recurrence, and risk factors. Thus, it is important to conduct a study even in developing countries like Nepal, where rising obesity has become an emerging problem.^6^

The aim of this study is to find out the prevalence of obesity in inguinal hernia repair patients in a tertiary care center.

## METHODS

This is a descriptive cross-sectional observational study performed on 219 hospitalized inguinal hernia patients at the Department of general surgery, Bir hospital, Kathmandu, Nepal, from May 2018 to December 2019. Ethical approval was obtained from the Institutional Review Board (IRB) of NAMS.

All Patients ≥15 years, clinically diagnosed cases of inguinal hernia, undergoing either elective or emergency surgery irrespective of the mode of anesthesia were included in the study.

Patients with inguinoscrotal swellings other than an inguinal hernia, who did not give consent, and patients with familial collagen vascular diseases were excluded from the study.

Total sample size was calculated by using


$$\begin{array}{ll} \text{n} & ={\text{Z}}^{\text{2}}\times \text{p}\times \text{q}/{\text{e}}^{\text{2}}\\ & ={\left(1.96\right)}^{2}\times 0.172(1-0.172)/{\left(0.05\right)}^{\text{2}} \end{array}$$

Where,

n = sample size
z = confidence interval (1.96 for Confidence Interval of 95%)
p = prevalence (17.2% i.e. 0.172)
q = 1-p
e = margin of error (5% i.e. 0.05)

After calculation, n= 218.8≈219 cases. Hence, taking the largest incidence, the required (largest) sample size for the study is 219 cases.

The patients were grouped into different BMI groups based on the WHO Asia-Pacific obesity classification as:

a) Underweight (body mass index, BMI: <18.5 kg/m^2^),
b) Normal (BMI ≥18.5 kg/m^2^ <22.9 kg/m^2^),
c) Overweight (BMI ≥23 kg/m^2^ <24.9 kg/m^2^),
d) Obese (BMI ≥25 kg/m^2^ to <29.9 kg/m^2^), and
e) Severely obese (BMI ≥30 kg/m^2^) by BMI.^7,8^

According to the BMI, patients higher than 23 kg/m^2^ were defined as overweight and patients between 18.5 kg/m^2^ and 22.9 kg/m^2^ were defined as normal-weight patients.

Heavy work was defined as lifting more than 100 pounds (45.3 kg) at a time with frequent lifting or carrying of objects weighing up to 50 pounds (23 kg).^9^ Chronic cough was defined as cough more than 3 months durations irrespective of phlegm.^10^

All eligible candidates were subjected to IHR and per-operative findings, difficulties, and complications were noted and correlated with the BMI. Participants were followed up for one year. Follow-up in 3 weeks and three months was done by patients visiting themselves in OPD and 12 months follow-up by either themselves visiting in OPD or via telephone inquiries regarding any complications.^11^

Statistical analysis was done using SPSS version 23 and Microsoft Excel software by descriptive statistics.

## RESULTS

Out of 350 patients, 219 were eligible for the study. Among them, 208 (95%) were male and 11 (5%) were female. The mean age was 51.69±8.52 years, weight 56.68±9.39 Kg, height 160.06±8.72 cm and BMI 22.10±3.07 kg/m^2^. BMI Category 18.5-22.9 kg/m^2^ has maximum number 141 (64.38%). One hundred and thirty four (61%) male and 7 (3.2%) female patients, and BMI category ≥30 kg/m^2^ has minimum number 4 (1.8%) male and none of the female.

Highest IHRs done in BMI group 18.5-22.9 kg/m^2^ 141 (64.2%) and lowest in ≥30 kg/m^2^ 4 (1.8%). The prevalence of IHR decreased as the BMI category increased and vice versa (Table 1).

**Table 1:** Distribution of BMI among IHR patients.

BMI Category (kg/m^2^)	Male n (%)	Female n (%)	Total n (%)
18.5-22.9	134 (61.18)	7 (3.19)	141 (64.38)
23-24.9	41 (18.72)	3 (1.36)	44 (20.09)
25-29.9	29 (13.24)	1 (0.45)	30 (13.69)
≥30	4 (1.82)	0 (0)	4 (1.82)
Total	207 (94.5)	11 (5.02)	219 (100)

**Figure 1.**
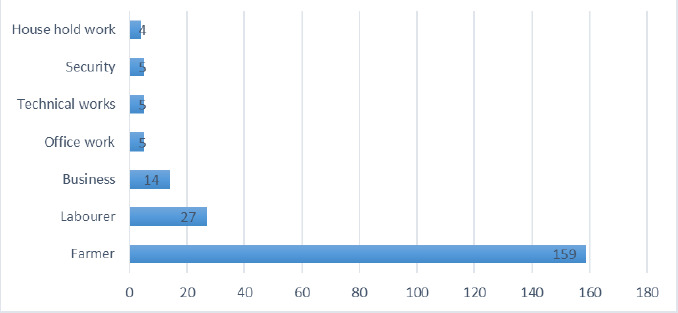
Shows the prevalence of IHR in different occupation groups.

Most of IHR patients were farmers 159 (72.5%) followed by laborers 27 (12.32%) and businessman 14 (6.39%) (Figure 1).

**Figure 2.**
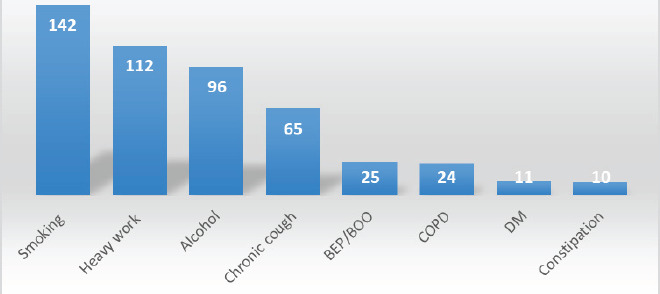
Risk Factors for Inguinal Hernia

Common risk factors responsible for the IHs were: smoking 143 (65.29%), chronic cough 65 (29.68%), heavy work 112 (51.14%), COPD 24 (10.95%), BEP/BOO 25 (11.41%) (Figure 2).

The distribution of primary and recurrent IHR in different BMI groups. Primary IH was more common in the low BMI category, while recurrent IHR increased as the BMI increased (Table 2).

**Table 2:** Distribution of BMI for Primary Vs Recurrent Hernia Repairs.

BMI category kg/m^2^	Primary n (%)	Recurrent	Total
18.5-22.9	135 (95.7)	6 (4.25)	141
23-24.9	42 (95.5)	2 (4.5)	44
25-29.9	27 (90.0)	3 (10.0)	30
=>30	4 (100.0)	0 (0)	4
	208 (94.9)	11 (5.02)	219

**Figure 3.**
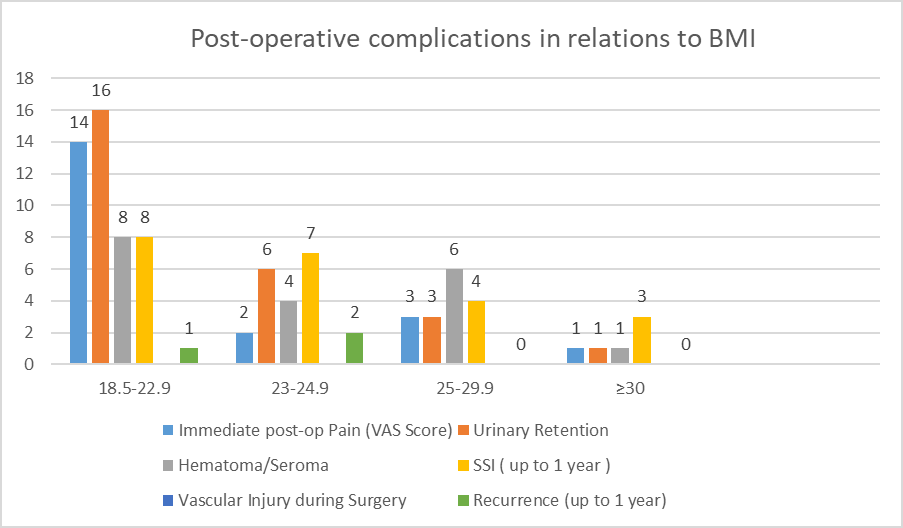
Post-operative complications in relation to BMI.

Most of the complications occurred in the normal and overweight BMI category and the prevalence of complications decreased as the BMI category increased except the Surgical Site Infection (SSI) (Figure 3). Recurrence was seen in 3 (1.4%) cases (one in open mesh repair, one in darning repair, and one in TAPP repair).

## DISCUSSION

In this study, most IHRs patients belonged to low BMI group, i.e., most IHR patients were not obese. This is similar to the study done by Zendejas et al., Ravanbakhsh et al., and Rosemar et al., which showed incidence and prevalence of groin hernia decreased as the BMI increased.^5,12,13^

Hypothesis in favor of obesity as a “protective” factor in the development of IH are:^12^

1) Intra-abdominal or pre-peritoneal fat acting as a “plug,”
2) Poor health conditions of the obese patient making them unfit for an elective operation such as an IHR,
3) Physical inactivity of the obese patient, though the degree and type of physical activity is a controversial risk factor for the development of an IHs and
4) Difficulty in clinical diagnosis and self-awareness in obese individuals due to body habitus.

As BMI reflects overall body weight rather than increased intra-abdominal pressure, other measures such as waist diameter and intra-abdominal visceral fat may be taken to prove or disprove the hypothesis.^14^

When compared with the iceberg analogy, the tip represents to surgery of IH (i.e., IHRs), and the portion under the waterline corresponds to both patients with a clinical diagnosis (physical examination, imaging, or self-awareness) and patients who are unaware of their IH. We decided to focus on the tip of the iceberg (i.e., IHRs, IH that were repaired) to observe the IHR and BMI distribution in the study population.

Most of the patients were farmers 159 (72.6%), showing the influence of the work. It increases the IAP and protrusion of the abdominal contents from the defects and the abdominal wall's weakest points. Smoking, heavy work and chronic cough were other common causes of IHs. A similar study done by Balamaddaiah G et al. and Carbonell et al. showed the most common cause for the presence of hernia was lifting heavy objects.^15,16^

Most of IHRs 208 (94.9%) were primary and 11 (5.02%) were recurrent. The recurrence was found in three cases (1.4%), which is much lower as compared to the study done by Sorensen et al. and Cheong et al., the recurrence rate was 12.7% (10.0-15.8) and 3.8% patients, respectively.^17,18^ Several factors that influence the recurrence were contaminated wounds, female gender and complexity of hernia.^19,20^

Most of the complications occurred in the normal and overweight BMI category and the frequency of complications decreased as the BMI category increased. However, when compared within the groups with increasing BMI, SSI prevalence increased. It is similar to a study done by Cheong et al.^18^

The major limitations of the study were short follow-up periods, small sample size, heterogeneous group of patients, no randomizations in the sampling and need for population-based study.

## CONCLUSIONS

The majority of IHR patients were non-obese (in other words, obesity is less prevalent in inguinal hernia patients). The increase in BMI leads to an increased intra-abdominal cause that may not cause the development of IH as we are thinking for years. Complications were common in non-obese IHRs patients except for the SSI. Nonetheless, further studies examining measures of obesity are required.
